# Supplementary material for: Development and comparison of single FLT3-inhibitors to dual FLT3/TAF1-inhibitors as an anti-leukemic approach
Source: PLoS One. 2025 Mar 28;20(3):e0320443. doi: 10.1371/journal.pone.0320443 (PMC11952222; doi:10.1371/journal.pone.0320443)
Supplement: S1 Table — Inhibition constant (IC50) of novel 3i-compounds against FLT3 and TAF1(2) (kdELECT, Eurofins/DiscoverX, USA). (PDF) [file pone.0320443.s001.pdf]

**S1 Table.** Molecular structures, tyrosine kinase- and bromodomain inhibition of compounds synthesized by University of Helsinki and Enamine (Kyiv, Ukraine). Inhibition constant ($IC_{50}$) of novel compounds against FLT3 and TAF1(2) (kdELECT, DiscoverX, USA).

| Code | Molecular structure | Molecular weight | FLT3 $IC_{50}$ (nM) | TAF1(2) $IC_{50}$ (nM) | Supplier (code) |
|---|---|---|---|---|---|
| **3i-1103** | 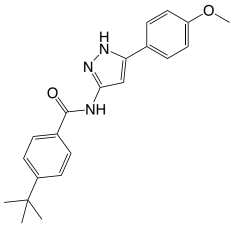 | 349 Da | <1000 | 480 | Faculty of Pharmacy Univ. of Helsinki Jumppanen *et al.* 2019 |
| **3i-1244** | 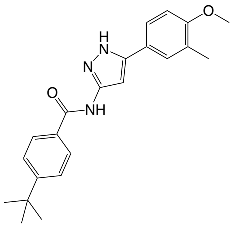 | 363 Da | <1000 | >10000 | Enamine Kyiv, Ukraine Z3159646172 |
| **3i-1245** | 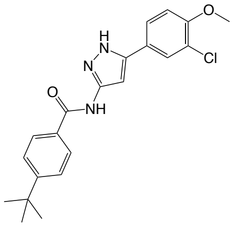 | 383 Da | <1000 | >10000 | Enamine Kyiv, Ukraine Z3159650063 |
| **3i-1246** | 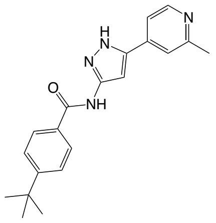 | 334 Da | 18 | 260 | Enamine Kyiv, Ukraine Z3159653200 |
| **3i-1247** | 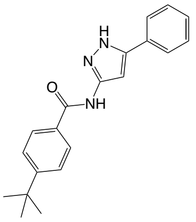 | 319 Da | 1.5 | >10000 | Enamine Kyiv, Ukraine Z3159687215 |
| **3i-1248** | 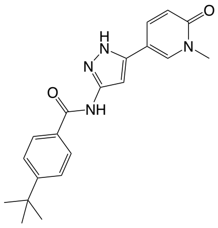 | 350 Da | <1000 | 5.1 | Enamine Kyiv, Ukraine Z3159666854 |
