# Supplementary material for: Development and comparison of single FLT3-inhibitors to dual FLT3/TAF1-inhibitors as an anti-leukemic approach
Source: PLoS One. 2025 Mar 28;20(3):e0320443. doi: 10.1371/journal.pone.0320443 (PMC11952222; doi:10.1371/journal.pone.0320443)
Supplement: S4 Fig — Dose response curves were generated in triplicate, and each dose-response curve represents an independent experiment. Dots represent individual data points and the gray area represents the 95% confidence interval for each curve. Both single FLT3 inhibitors (3i-1244, 3i-1245, 3i-1247) and dual FLT3/TAF1 inhibitors (3i-1103, 3i-1246, 3i-1248) were used for experiments. Compounds for which IC50 values could not be generated are listed as n.d. (not determined). (PDF) [file pone.0320443.s005.pdf]

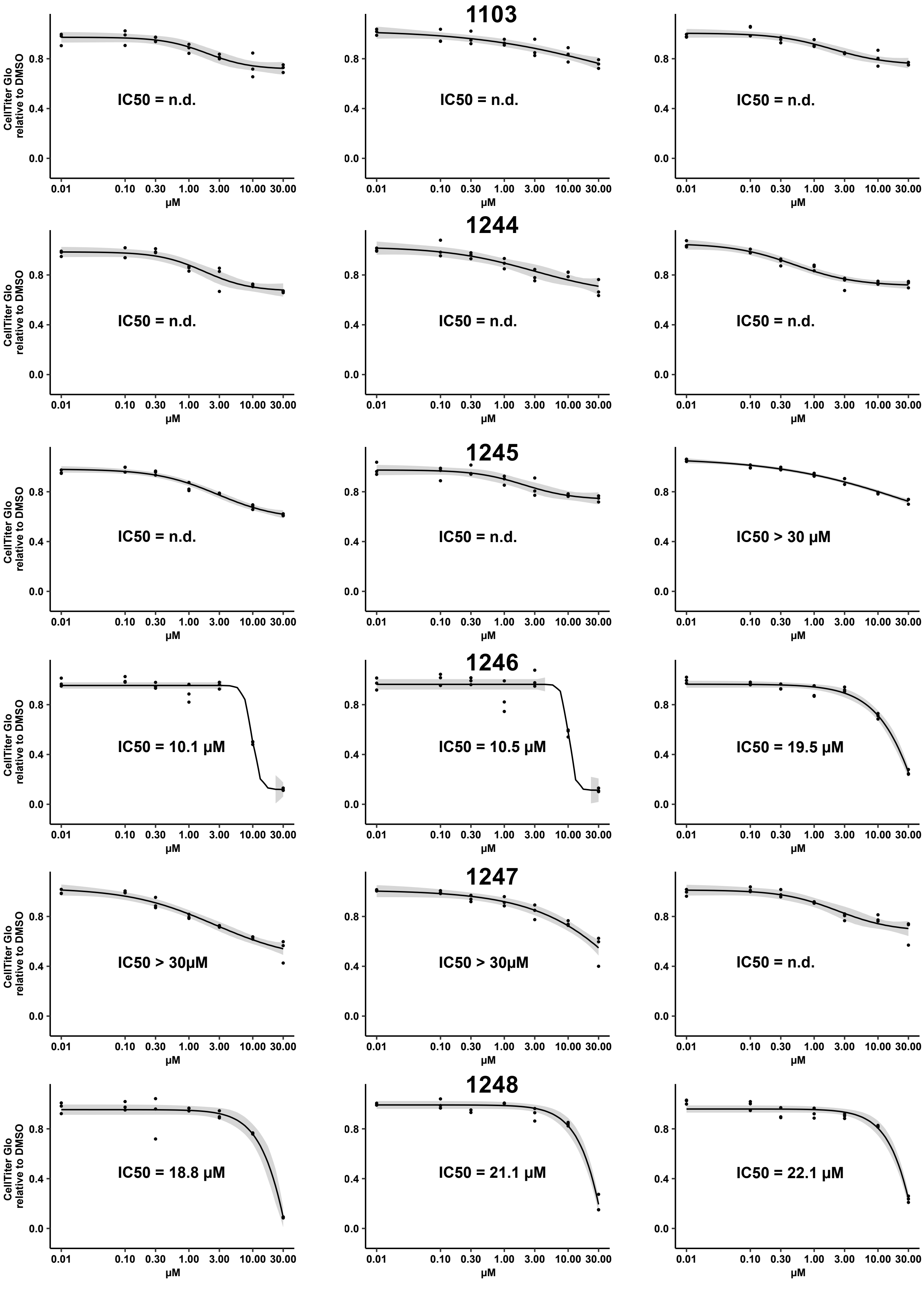

Supplementary Figure S4. Anti-oncogenic activity of novel compounds and reference drugs *in vitro.* HDQP1 cells were treated with a series of FLT3/TAF1 compounds and cell viability was determined using a CellTiter Glo assay. Dose response curves were generated in triplicate, and each dose-response curve represents an independent experiment. Dots represent individual data points, and the gray area represents the 95% confidence interval for each curve. Both single FLT3 inhibitors (1244, 1245, 1247) and dual FLT3/TAF1 inhibitors (1103, 1246, 1248) were used for experiments. Compounds for which IC50 values could not be generated are listed as n.d. (not determined).
