# Supplementary material for: Development and comparison of single FLT3-inhibitors to dual FLT3/TAF1-inhibitors as an anti-leukemic approach
Source: PLoS One. 2025 Mar 28;20(3):e0320443. doi: 10.1371/journal.pone.0320443 (PMC11952222; doi:10.1371/journal.pone.0320443)
Supplement: S5 Fig — (A) Compound activity in IGROV1 cells at 0.01-30 µM (n ≥ 3). (B) pIC50 and IC50 values of FLT3/TAF1 inhibitors. Dots represent independent experiments (n = 3) and mean ± SEM is shown. Both single FLT3 inhibitors (3i-1244, 3i-1245, 3i-1247) and dual FLT3/TAF1 inhibitors (3i-1103, 3i-1246, 3i-1248) were used for experiments. Compounds for which IC50 values could not be generated for three experiments or which were determined to be greater than the maximal tested concentration (30 µM) are listed as n.d. (not determined). (PDF) [file pone.0320443.s006.pdf]

A

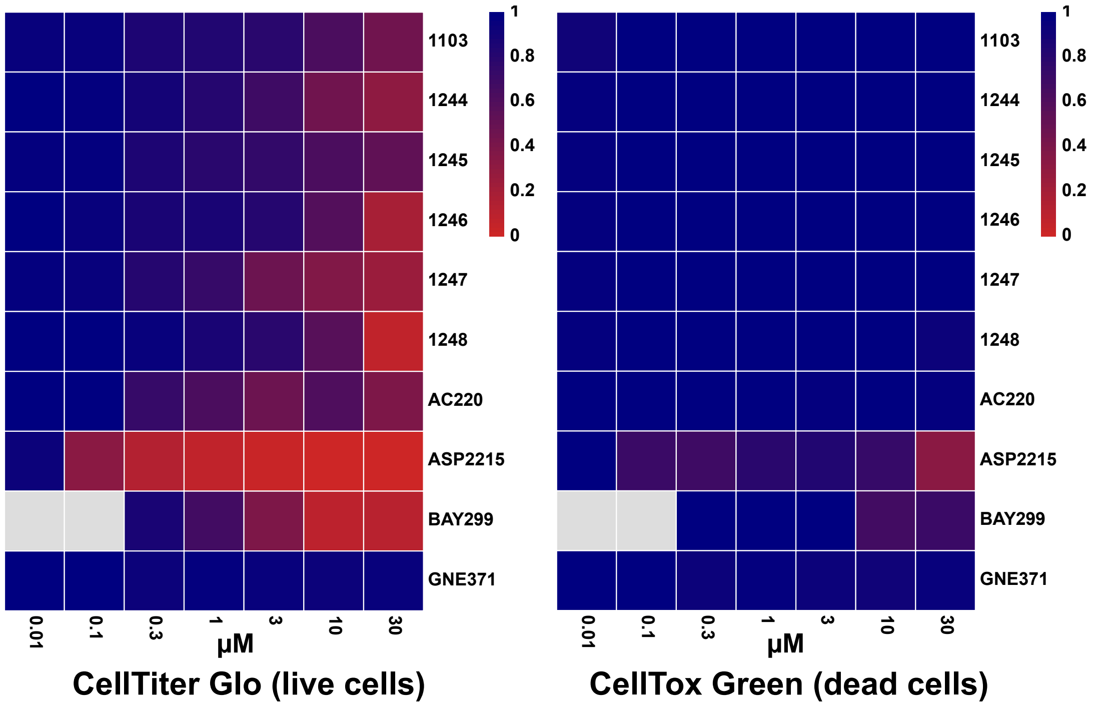

B

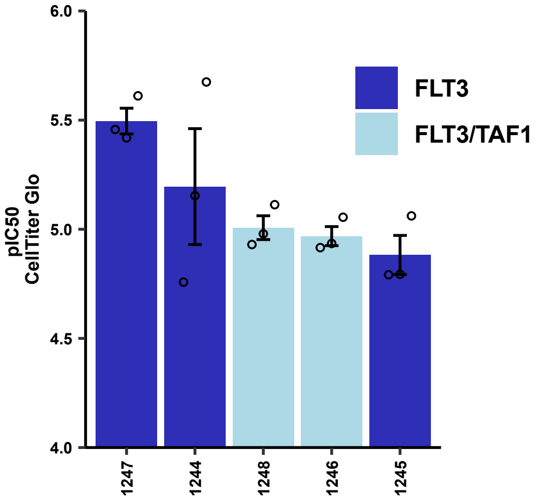

IGROV1

Compound	IC50	Target
1247	3.3 µM	FLT3
1244	8.9 µM	FLT3
1248	10 µM	FLT3/TAF1
1246	10.9 µM	FLT3/TAF1
1245	13.6 µM	FLT3
1103	n.d.	FLT3/TAF1

Supplementary Figure S5. Anti-oncogenic activity of novel compounds and reference drugs in vitro. IGROV1 cells were treated with a series of FLT3/TAF1 compounds and cell viability was determined using a Cell-titer Glo assay, while cell toxicity was determined using a CellTox Green assay. **A** Compound activity in IGROV1 cells at 0.01-30 µM ($n \geq 3$). **B** pIC_{50} and IC_{50} values of FLT3/TAF1 inhibitors. Dots represent independent experiments (n=3) and mean ± SEM is shown. Both single FLT3 inhibitors (1244, 1245, 1247) and dual FLT3/TAF1 inhibitors (1103, 1246, 1248) were used for experiments. Compounds for which IC50 values could not be generated for three experiments or which were determined to be greater than the maximal tested concentration (30µM) are listed as n.d. (not determined).
