# Supplementary material for: Development and comparison of single FLT3-inhibitors to dual FLT3/TAF1-inhibitors as an anti-leukemic approach
Source: PLoS One. 2025 Mar 28;20(3):e0320443. doi: 10.1371/journal.pone.0320443 (PMC11952222; doi:10.1371/journal.pone.0320443)
Supplement: S7 Fig — (A) Compound activity in KASUMI-1 cells at 0.001-30 µM (n = 1). (B) IC50 values of FLT3/TAF1 inhibitors. Each dose-response curve represents an independent experiment. Dots represent individual data points and the gray area represents the 95% confidence interval for each curve. Both single FLT3 inhibitors (3i-1244, 3i-1245, 3i-1247) and dual FLT3/TAF1 inhibitors (3i-1103, 3i-1246, 3i-1248) were used for experiments. (PDF) [file pone.0320443.s008.pdf]

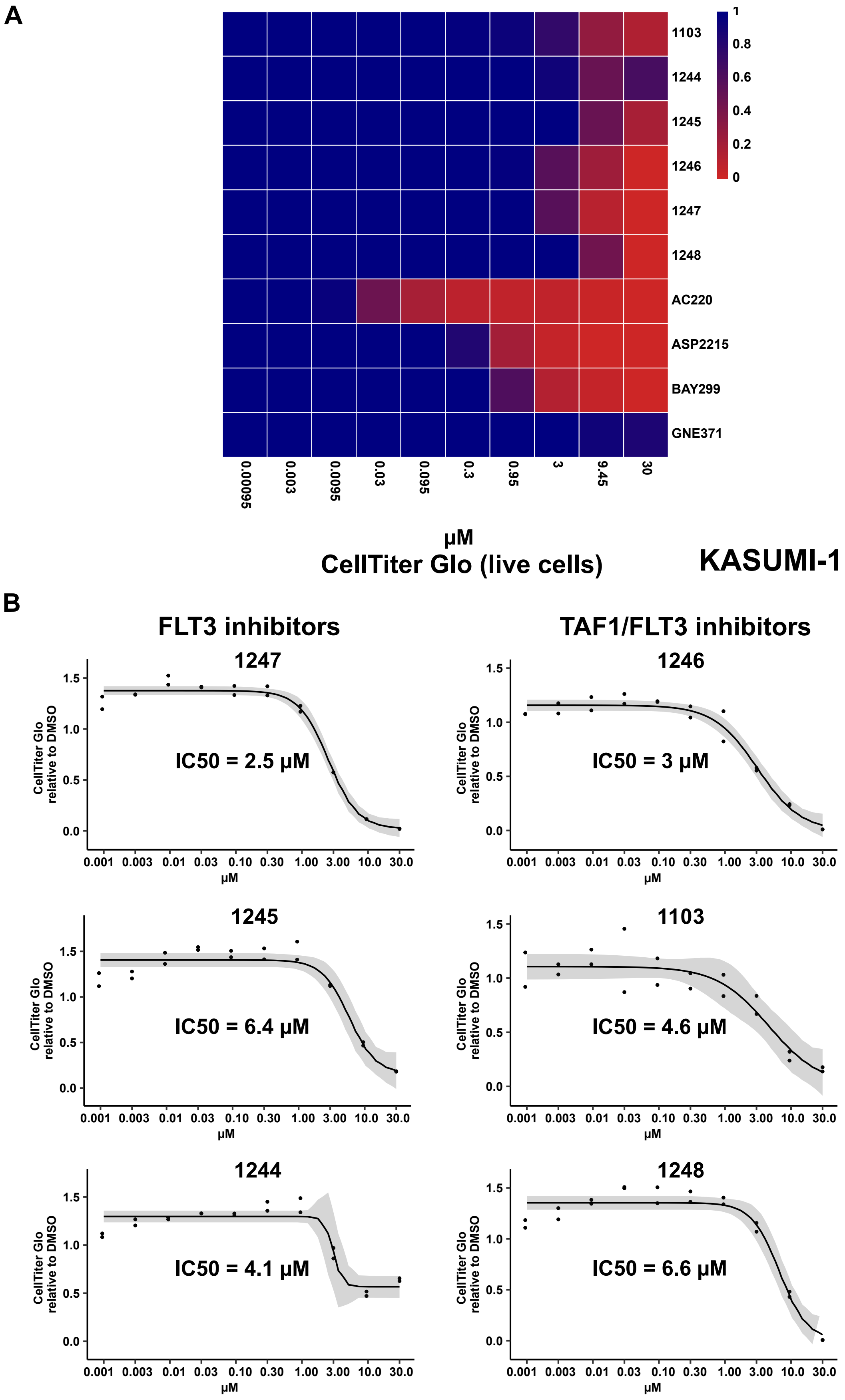

Supplementary Figure S7. Anti-oncogenic activity of novel compounds and reference drugs *in vitro.* KASUMI-1 cells were treated with a series of FLT3/TAF1 compounds and cell viability was determined using a CellTiter Glo assay, while cell toxicity was determined using a CellTox Green assay. **A** Compound activity in KASUMI-1 cells at 0.001-30 µM (n = 1). **B** IC_{50} values of FLT3/TAF1 inhibitors. Each dose-response curve represents an independent experiment. Dots represent individual data points, and the gray area represents the 95% confidence interval for each curve. Both single FLT3 inhibitors (1244, 1245, 1247) and dual FLT3/TAF1 inhibitors (1103, 1246, 1248) were used for experiments.
